# Evidence of a tolerogenic vaccine against AIDS in the Chinese macaque prefigures a potential human vaccine

**DOI:** 10.1007/s00705-020-04935-6

**Published:** 2021-01-28

**Authors:** Jean-Marie Andrieu, Wei Lu

**Affiliations:** 1 Laboratory of Autoimmunity and Inflammation, Cochin Institute, Université de Paris, 75013 Paris, France; 2 Institut de Recherche sur les Vaccins et l’Immunothérapie des Cancers et du SIDA, Centre Universitaire des Saints Pères, Université de Paris, 75006 Paris, France; 3 Institut de Recherche pour le Développement (IRD), 13000 Marseille, France

## Abstract

In 2006 we discovered a new type of mucosal vaccine against simian immunodeficiency virus (SIV) in Chinese macaques. Here, we review 15 years of our published work on this vaccine, which consists of inactivated SIVmac239 particles adjuvanted with Bacillus Calmette-Guérin, *Lactobacillus plantarum*, or *Lactobacillus rhamnosus*. Without adjuvant, the vaccine administered by the intragastric route induced the usual SIV-specific humoral and cellular immune responses but provided no protection against intrarectal challenge with SIVmac239. In contrast, out of 24 macaques immunized with the adjuvanted vaccine and challenged intrarectally with SIVmac239 or SIVB670, 23 were sterilely protected for up to five years, while all control macaques were infected. This protection was confirmed by an independent group from the Pasteur Institute. During the past 15 years, we have identified the mechanism of action of the vaccine and discovered that the vaccinated macaques produced a previously unrecognized class of MHC-Ib/E-restricted CD8^+^ T cells (which we refer to as tolerogenic CD8^+^ T cells) that suppressed the activation of SIV-RNA-infected CD4^+^ T cells and thereby inhibited the (activation-dependent) reverse transcription of the virus, which in turn prevented the establishment of SIV infection. Importantly, we discovered also that the tolerogenic CD8^+^ T cell subset observed in vaccinated Chinese macaques could also be found in human elite controllers, a small group of HIV-infected patients in whom these tolerogenic CD8^+^ T cells were shown to naturally suppress viral replication. Given that SIV and HIV require activated immune cells in which to replicate, the specific prevention of activation of SIV-RNA-containing CD4^+^ T cells by a tolerogenic vaccine approach offers an exciting new avenue in HIV vaccine research.

## Introduction

Human immunodeficiency virus (HIV) infection is a chronic retroviral infection that is principally transmitted by sexual contact. The virus entering the genital mucosa replicates in its preferred target cell, the activated CD4^+^ T cell, a centerpiece of the immune system. In the absence of antiviral treatment (one that suspends HIV replication as long as it is taken), the chronic infection induces a slow and clinically occult destruction of the immune system, ending for more than 99% of infected people in fatal infections and tumors after a median of 8-9 years. During these years, untreated infected individuals (most of whom are not aware of their infection) are spreading the disease by sexual transmission. The result is that, despite the ambitious program of HIV detection and treatment proposed by UNAIDS [1], the epidemic is still spreading throughout the world. In 2019, 38 million people were living with the infection, 1.7 million were newly infected, and 690,000 died from the infection [2]. Because the genital mucosa is the site where the infection begins, our objective was to develop a mucosal vaccine capable of arresting the virus at the mucosal border. For that purpose, we used a Chinese rhesus macaque (ChRM) vaccine model challenged with the simian immunodeficiency (SIV) strain mac239, which is a viral clone commonly used in rhesus macaque models of HIV transmission and pathogenesis because it induces high viral loads and AIDS progression.

In this narrative literature review, we first summarize recent results of human and macaque vaccine trials. Then, we recall some features of the early HIV/SIV infectious process that are important for understanding the tolerogenic mechanism of our vaccine. The main part of the review is then devoted to several studies we have conducted and published on the efficacy, longevity, and mechanisms of action of this new type of vaccine. Its protective efficacy against SIV challenge, together with the concomitant identification of a robust *ex vivo* correlate of protection, suggests a new approach for developing a preventive HIV vaccine for humans. Details on the methods and results analyzed in this review are available in the original publications [3–6]. The figures are also adapted from the original publications. We would like to alert the readers that some paragraphs in this review appeared in a previously published research article we wrote [54].

## HIV vaccine

### Vaccine efficacy in 2020 human vaccine trials

In the face of this disastrous epidemic, the discovery of a safe and effective vaccine remains the only solution to curb the number of new infections [7]. The current paradigm in the field of HIV vaccinology is still that a vaccine must generate, first and foremost, high and durable titers of broadly neutralizing HIV-envelope-specific antibodies capable of neutralizing the entering virus before it infects the first mucosal target CD4^+^ T cell encountered (while the role of cellular immune responses is still being debated in the setting of preventive vaccines). Taking into consideration the profound variability of the HIV genome in human populations and particularly the variability of the viral envelope gene, which encodes a protein that is readily accessible to the immune system, vaccinologists have so far not identified the right recombinant proteins or the right combination of viral genes encoding proteins that would elicit the production of high-affinity antibodies capable of neutralizing HIV entering the mucosa. This is illustrated by the results of human vaccine efficacy trials [8].

The clinical trials Step /HVTN 502 (2004), Phambili/HVTN 503 (2005), and HTVN 505 (2009) were aimed at testing replication-defective adenovirus vectors expressing HIV-1 genes. Unexpectedly, the results of these trials showed an enhanced acquisition of HIV infection in the randomized groups that received the vaccine. These observations (which led to the premature interruption of the second and third trials) raised the possibility that vaccine stimulation of activated vector-specific or even total CD4^+^ T cells present in the mucosa may lead to enhanced HIV acquisition [9, 10]. Such increased mucosal CD4^+^ T cell activation following vaccination with an adenoviral vector was also demonstrated in the rhesus macaque (RM) model [11, 12]. Finally, although a canarypox vector and gp120 regimen was partially efficacious at 3 years post-vaccination in a modified intention-to-treat analysis in Thailand [13], it did not show statistically significant efficacy in the per-protocol or intent-to-treat analyses. Furthermore, when adapted for subtype C, there was no efficacy in South Africa [14].

### Vaccine trials in rhesus macaques

Although the ChRM model was most likely the best model to test preventive vaccines [15], the vast majority of pre-clinical NHP studies were performed in the Indian rhesus macaque (InRM) model. A careful review of the literature of the last 35 years indicates that, after having excluded the numerous vaccine prototypes that induced protection against artificial viral challenge with simian-human immunodeficiency virus (SHIV), there remained only one vaccine prototype that was capable of inducing (partial) protection against the "neutralization-resistant" SIVmac251. This vaccine consisted of an adenovirus 26 vector expressing SIV Env/Gag/Pol antigens prime boosted with SIV Env gp140 adjuvanted with AS01B. After six intrarectal challenges with 500 TCID_50_ of SIVmac251, sterile post-challenge protection was observed in 50% of the vaccinated InRMs. Importantly, the protective efficacy correlated with the functionality of envelope-specific antibody responses [16]. A similar HIV vaccine regimen, but with a different adjuvant, was injected intramuscularly into humans. The immune response results of these early-phase human trials showed that both systemic and mucosal HIV-1-Env-specific humoral and cellular responses had been elicited in the majority of subjects [17, 18]. A multi-center phase 3 trial (MOSAICO) is presently ongoing to test the efficacy of this vaccine.

## The infectious process

The primary routes of infection by HIV are sexual and anal (as well as oral in newborns). These mucosal routes of infection are reproducible with SIV in the RM model. The first step of the infectious process is entry of the mucosa. It is now widely agreed that only the "transmitter/founder" HIV particles (or SIV in RMs) that bear some specific envelope “signatures" can cross the epithelial barrier of the genital mucosa [19, 20]. Once the transmitter/founder virus has penetrated the mucosa, it makes contact with the first target cell encountered, a CD4^+^ T cell*.* Two phenomena then occur simultaneously. First*,* virus attachment to a CD4 receptor and a CCR5 co-receptor is followed within a short time (2 hours) by the presentation at the plasma membrane of Gag and Pol antigens derived from incoming virions [21]; this transforms the CD4^+^ T cell into a Gag/Pol^+^CD4^+^ T cell, while Env and Nef proteins need *de novo* synthesis, i.e. a round of viral replication in the target cell [22]. On the other hand, at the same time, viral RNA enters the target Gag/Pol^+^CD4^+^ T cell. The destiny of the infection is now at stake, depending on the activation status of the HIV-RNA-infected Gag/Pol^+^CD4^+^ T cell. If it is in a quiescent/non-activated state (which is the usual situation in non-high-risk groups of Western countries, where chronic genital infection or inflammation is infrequent), HIV RNA reverse transcription is very inefficient or abortive [23–25], and the infectious process is arrested. In contrast, if the HIV-RNA-infected, Gag/Pol^+^CD4^+^ T cell is in an activated state (which may occur more frequently in the inflammatory/infectious environment that may be observed in tropical areas, and even more in high-risk groups), the HIV RNA that has penetrated the Gag/Pol^+^CD4^+^ T cell is easily reverse transcribed into HIV DNA. This step is then irreversibly followed by a cascade of cellular events ending in the production and release of large amounts of free viral proteins and defective virions (which activate surrounding immune cells) as well as HIV virions, which now replicate easily in these activated CD4^+^ T cells. Very quickly, infectious CD4^+^ T cells as well as free viral particles spread throughout the organism. This is in keeping with the notion that the early activation of a small population of infected CD4^+^ T cells at the portal of entry is required for the local expansion and establishment of systemic infection [26, 27]. Within a week, virus-specific CTLs are produced, followed by antiviral antibodies. However, at this point, it is too late, since many viral proteins (including the viral envelope) have already mutated and have thereby escaped both CTL killing and antibody neutralization. Such an ineffective immune response allows HIV infection to perpetuate in a chronic manner. The same steps are observed with SIV in the RM model.

## Tolerogenic vaccine against HIV: a new paradigm

Generally, the HIV vaccines that have been tested were intended to induce immune activation, which in some cases unwittingly increased the number of HIV target cells and therefore increased susceptibility to infection. A different approach to inducing immune activation is to use a vaccine that suppresses the HIV-specific immune response, so as to decrease the number of HIV targets, and this approach is called "tolerogenic vaccination”, as has been described for multiple sclerosis [28].

### BCG, a tolerogenic mucosal vaccine adjuvant discovered by serendipity

At the end of the 1990s, we had explored the capacity of blood-monocyte-derived dendritic cells (DCs) loaded with chemically inactivated (killed) HIV to stimulate HIV-specific cellular immunity *in vitro* [29]. In the following years, we showed that (subcutaneous) therapeutic vaccines comprising inactivated SIV- or HIV-loaded DCs had a favorable impact on SIV and HIV replication in infected ChRMs and humans, respectively [30, 31]. By the end of 2005, we decided to enter the research field of preventive SIV/HIV vaccines. Since HIV is mainly transmitted by mucosal contact, our first interest was to build a mucosal vaccine. Instead of activating DCs by loading them *ex vivo* with inactivated virus (like in our above-mentioned therapeutic vaccines), we thought it would be more interesting to produce a vaccine that would directly stimulate mucosal DCs *in vivo.* We thus prepared an inactivated SIVmac239 (iSIV) vaccine identical to the one we used previously in RM therapeutic vaccines. However, to amplify the ability of this vaccine to stimulate mucosal DCs, we thought that Bacillus Calmette-Guérin (BCG) could be an interesting adjuvant. BCG is an attenuated bovine-tuberculosis-bacterium-based vaccine against human tuberculosis that was administered safely for more than 90 years to more than 3 billion people until it finally became clear that it did not confer reliable and consistent protection against tuberculosis, except in its severe forms in children [32]. BCG was also given to several million infants via the mucosal (oral) route in Brazil until 1974 without serious side effects [33]. We chose BCG as an adjuvant of our mucosal vaccine because it had been shown to strongly stimulate mucosal DCs/Langerhans cells [34, 35]. This characteristic was particularly useful for treating superficial bladder cancer against which BCG was (and still is) given by direct mucosal bladder instillation [36]. We proposed the hypothesis that, if given by the mucosal route together with our iSIV vaccine, BCG, by stimulating mucosal DCs/Langerhans cells, would activate the Th1 lymphocyte pathway, which would in turn favour cellular responses against SIV-infected cells. We chose the ChRM model to test our experimental vaccines because it is genetically closer to humans than the InRM model [37–41]. We chose the vagina as the first site of mucosal immunization because it is the principal site of heterosexual transmission.

In early 2006, seven ChRMs were administered a vaccine intravaginally comprising iSIV adjuvanted with BCG (strain SSI 1331). A booster intravaginal immunization with similar doses was applied two months later. Five control RMs were given BCG alone according to the same protocol. At four months post-immunization, the 12 (7 vaccinated + 5 controls) RMs were challenged by the intrarectal route with a high infectious dose of SIVmac239 (100,000 TCID_50_). The result of this challenge was surprising: four out of the seven vaccinated ChRMs became and remained sterilely protected with undetectable levels of plasma SIV RNA and cellular proviral DNA over the next 60 days of the study. The three remaining vaccinated RMs showed a mild SIV RNA peak followed by low viral RNA and DNA load setpoints, while the five control animals showed typical primary infection [4].

In view of these very unusual findings, we decided to test whether our vaccine would work in the same manner when administered by another mucosal route. We chose the gastric route because, if successful, it could prefigure a human oral vaccine. The vaccine containing iSIV and BCG was thus administered through a gastric tube to four RMs. Booster vaccinations with the same preparation were repeated at days 30 and 60, and four control RMs were given BCG alone. By day 90, a massive intrarectal challenge with SIVmac239 (100,000 TCID_50_) was given to the eight animals. The four control RMs showed typical primary infection. In strong contrast, all four vaccinated RMs were sterilely protected over the following 60 days, suggesting that SIV infection was blocked at entry by mucosal immunity.

Moreover, when we tested the SIV-specific humoral and cellular immune responses of these RMs before challenging them, we had a new surprise: SIV-specific antibodies and SIV-specific interferon-γ-releasing T-cell responses were undetectable and remained absent post-challenge.

We were thus facing the unexpected picture of a mucosal vaccine consisting of iSIV adjuvanted with BCG that induced the suppression of SIV-specific humoral and cellular responses and at the same time provided sterile post-challenge protection.

Taking into account that SIV- (or HIV)-specific CD4^+^ T cell activation is the driving force of the initial burst of viral replication *in vivo* [42–44] and conversely, that the suppression of CD4^+^ T cell activation by local application of an anti-inflammatory agent has been shown to prevent mucosal SIV transmission [45], the only hypothesis that could reconcile the two above-mentioned apparently contradictory observations was that our vaccine had induced SIV-specific tolerance, i.e., the suppression/prevention of SIV- specific CD4^+^ T cell activation, which thereby prevented viral reverse transcription from operating [23–27].

### *Lactobacillus plantarum*, a tolerogenic adjuvant

**Fig. 1:**
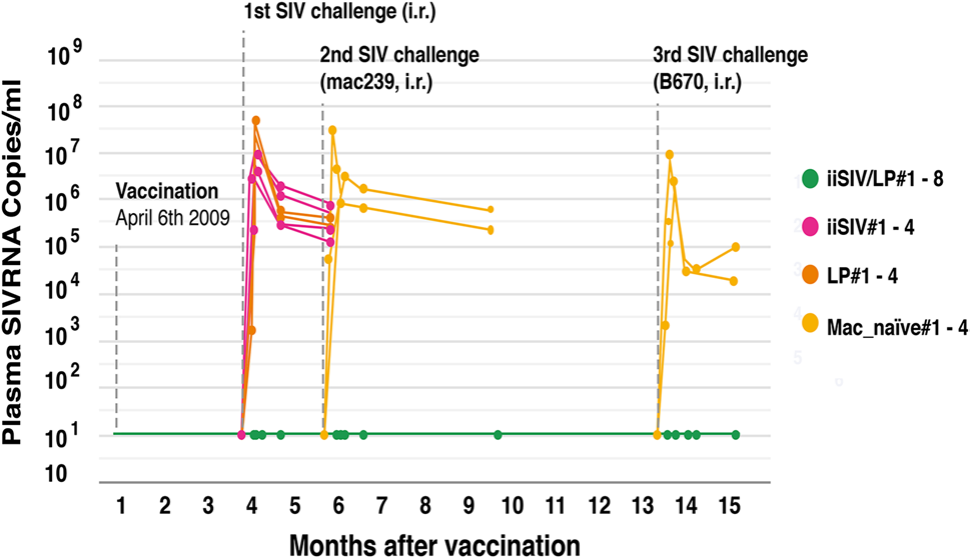
The evolution of the plasma SIV RNA load in ChRMs repeatedly receiving the iiSIV/LP vaccine. The eight vaccinated macaques were euthanized at month 15, and no trace of SIV DNA or RNA was found at autopsy

With the strong suspicion that it was the suppression of SIV-specific CD4^+^ T cell activation induced by the BCG-adjuvanted vaccine that had prevented the establishment of SIV infection, we decided, in place of BCG, to test another bacterial adjuvant, *Lactobacillus plantarum* (LP), a nonpathogenic intestinal commensal bacterium that had been suspected to induce some forms of tolerance [46–48]. It was in 2009 that we immunized, via the intragastric route, the first group of eight ChRMs; they received a vaccine comprising iSIV adjuvanted with LP, while four animals received LP only, and four received iSIV only. Before challenging the animals, we again observed that SIV-specific antibody and interferon-γ-producing cell responses were completely suppressed in the eight vaccinated RMs, while they were present in the four RMs intragastrically immunized with iSIV only. By day 90 post-immunization, the 16 animals were challenged intrarectally with 100,000 TCID_50_ of SIVmac239. Again, the eight RMs immunized with iSIV + LP were sterilely protected, while the eight control animals were infected. The sterile protection was associated with the suppression of SIV-specific humoral and classical cellular responses. The sterile protection was confirmed by the results of a second intrarectal challenge performed two months later in four of the eight already protected RMs. Moreover, the same four animals again remained fully protected against a third intrarectal challenge performed eight months later (i.e., 13 months after vaccination), this time with 100,000 TCID_50_ of the antigenically distinct SIVB670 strain (Fig. 1). This suggested that the intragastric vaccine administered to ChRMs was cross-protective, presumably through prevention of the activation of CD4^+^ T cells infected by another SIV strain [3].

### Long-term protection of vaccinated macaques verified by an external research group

To study the longevity of the protection, in March 2010 we administered our iSIV/LP vaccine through a stomach tube to eight new ChRMs, while four received iSIV only and the last four received LP only. A first intrarectal challenge was administered 14 months after vaccination, and we found that one vaccinated RM was not protected, while the other seven were again fully protected, as indicated by the absence of any SIV RNA or DNA in plasma and blood cells or in the rectal mucosa or pelvic lymph node lymphocytes. Pre- (and post-) challenge SIV-specific humoral and cellular responses were also suppressed in the seven successfully challenged RMs, while the four RMs immunized with iSIV alone developed usual plasma SIV-specific IgM and IgG and interferon-γ-producing cell responses.

**Fig. 2:**
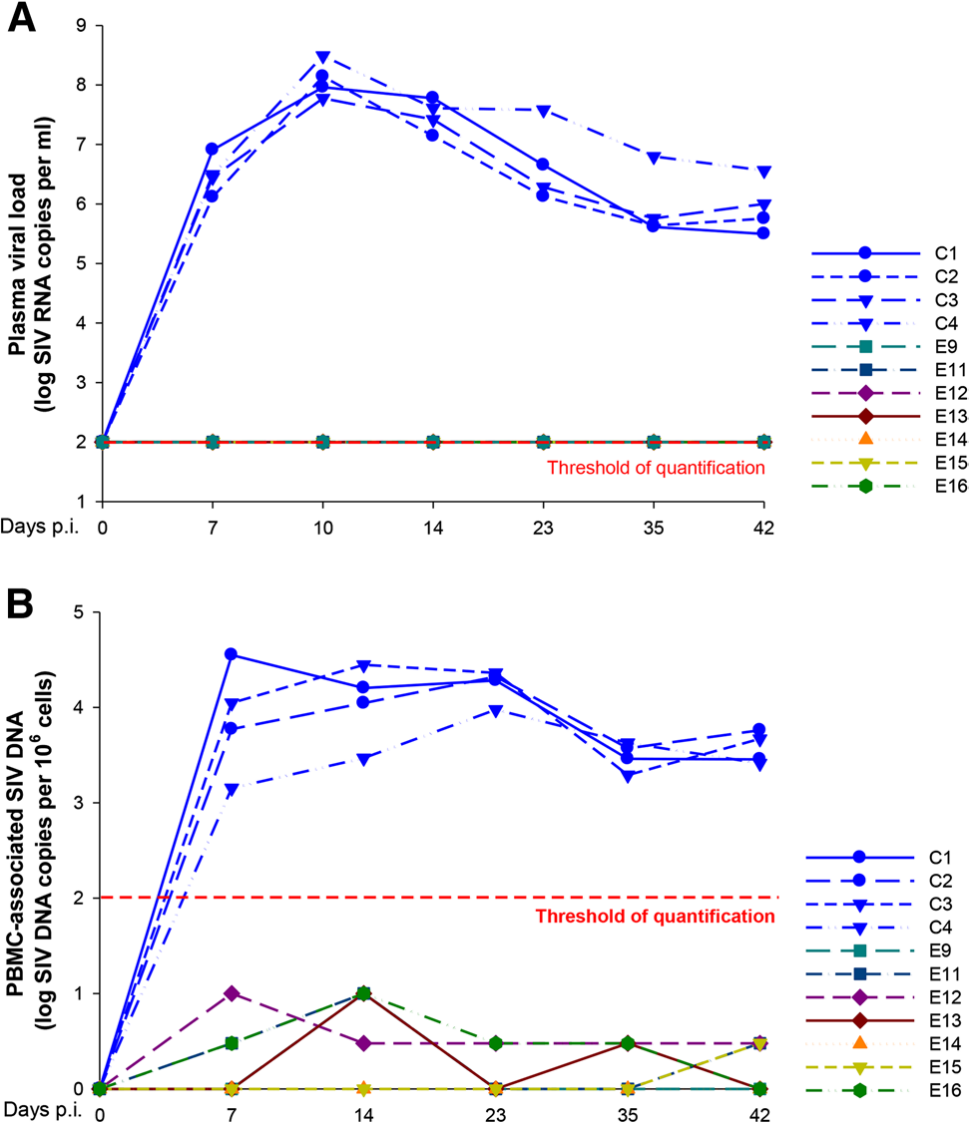
Plasma SIV RNA and PBMC-associated SIV DNA loads in control macaques (C1-C4) and in vaccinated macaques (E9, E11-E16) after challenge with SIVMac239. (A) All control animals became infected, and RNA levels increased exponentially, peaking at day 10 after infection and then decreasing gradually. No viral RNA was detected in the plasma of vaccinated animals. The quantification limit for this assay was 100 RNA copies/ml. (B) DNA levels in control animals were already over the quantification threshold at day 7 postinfection and increased in three animals until days 14-23. Six of the seven vaccinated animals had extremely low levels of viral DNA that were below the limit of quantification. In most cases, PCR was positive only in one out of two replicate wells, except for E9, E11, E13 and E16 at day 14 and E12 at day 7. Values corresponding to positive replicate wells were arbitrarily set to 10 copies, and values corresponding to only one positive well were arbitrarily set to 3 copies. The quantification limit of the assay was 100 DNA copies/ml.

Since this remarkable result actually corresponds to the best protective effect of an SIV vaccine observed so far, and because of its possible extension to humans, a complementary study was conducted, under the supervision of an independent specialist of the Institut Pasteur of Paris (Gianfranco Pancino) and approved by Stefano Marullo (as Vice President for Research of the University Paris Descartes), to establish whether the protection by the vaccine would be long lasting. Briefly, seven vaccinated monkeys from the original study, already protected against a first viral challenge, received a new intrarectal challenge of infectious SIVmac239 (10^5^ TCID_50_) 35 months and 21 months after the vaccination and the first challenge, respectively. Four naïve control monkeys were also infected using the same route. On days 7, 10, 14, 23, 35 and 42 after challenge, total RNA was extracted from plasma and DNA was extracted from freshly isolated PBMCs from vaccinated and control animals. Plasma SIV RNA and PBMC-associated SIV DNA loads were quantified by RT-qPCR and qPCR, respectively (Fig. 2). At day 0, both SIV RNA and DNA were undetectable in control and vaccinated monkeys. Starting at day 7, SIV RNA rose in the plasma of all control animals up to 5.9 × 10^7^-3.1 × 10^8^ copies/ml at day 10 post-challenge and then decreased progressively to 3.1 × 10^5^-3.6 × 10^6^ copies/ml at day 42 (Fig. 2A). In contrast, SIV RNA remained undetectable in all vaccinated monkeys throughout the experiment. PBMC-associated SIV DNA was detectable at day 7 and later on (up to 2.3-3.5 × 10^4^ copies per 10^6^ cells) in all control animals (Fig. 2B). It remained undetectable in one of the seven vaccinated animals, whereas extremely low levels of viral DNA were detected at some time points in the other six animals, falling below the accuracy level of the assay (Fig. 1B). Thus, vaccinated animals did not show any detectable viral replication after the second intrarectal challenge with SIVmac239 nearly 3 years after vaccination, indicating a remarkably long-lasting protection by the vaccine. Although only traces of viral DNA were detected in the PBMCs of six vaccinated monkeys, this finding suggests that the challenge with a very high viral inoculum may have initiated an infection in vaccinated animals that was subsequently turned down and controlled by the vaccine-induced immune response.

### Absence of immunoreactivity of Indian macaques to intragastric immunization with inactivated SIV

At the suggestion of Jose Esparza, then adviser for AIDS research at the Bill and Melinda Gates Foundation, Guido Silvestri and his team from Atlanta agreed to replicate our study, not in ChRms but in InRMs, although InRMs were known to be genetically more distant from humans than ChRMs [26, 27]. Inactivated SIVmac239 and LP were transferred from our labs to Atlanta. By the end of March 2015, 17 InRMs received the full vaccine (iSIV plus LP) intragastrically*,* ten received iSIV only, 10 received LP only, and 17 received a sham intragastric immunization. Intrarectal challenge with high doses of SIVmac239 performed three months later resulted in rapid infection in all groups of immunized InRMs as well as in the unvaccinated controls [5]. Importantly, the vaccine made of inactivated SIV particles given alone to 10 control InRMs did not induce an SIV-specific antibody response. In contrast, the same inactivated SIV particles administrated alone to a total of 10 ChRMs via the same intragastric route (in three separate experiments) were highly immunogenic, as shown by the SIV-specific humoral and cellular immune responses they generated. Taking into account that both ChRMs and InRMs are infected in the same manner with SIVmac239 or 251, it is highly surprising to observe the strong immunogenicity of iSIV particles given by the gastric route in ChRMs versus the complete absence of immunoreactivity to the same iSIV particles given by the same mucosal route in InRMs. Such a complete lack of a humoral and cellular response first suggests that, for whatever the reason, inactivated SIV particles did not reach the gastric mucosa. Alternatively, this strong and unexpected difference between InRM and ChRM immune reactivity toward ingested iSIV could have resulted from the genetic background of the two RM subspecies [26, 27]. In this setting, if the second hypothesis is the right one, InRMs are not an appropriate model to test vaccines given via the digestive route.

### Lactobacillus rhamnosus, a tolerogenic adjuvant

Because the *Lactobacillus plantarum* strain ATCC8014 had never been used in humans, we decided to test the commercial probiotic strain *Lactobacillus rhamnosus* (LR) (LCR35 from Biose, 15000 Aurillac, France) as a bacterial adjuvant. Four RMs were vaccinated intragastrically with iSIV and LR: two control RMs received LR only, and the other two received iSIV only. Twelve weeks post-vaccination, the eight RMs were administered 100,000 TCID_50_ of SIVmac239 intrarectally. The four vaccinated RMs were sterilely protected, while the four control RMs were infected. In total, we have administered a mucosal vaccine made of inactivated SIV particles adjuvanted with BCG, LP or LR to 31 RMs, of which 27 were found to be sterilely protected, while all control RMs got infected. In July 2015, all protected RMs were euthanized, and no trace of SIV RNA or DNA was found in the lymph nodes, spleen, G.I. tube, or brain of these animals.

## *Ex vivo* studies

### Suppression of SIV-specific CD4^+^ T cell activation by CD8^+^ T cells

**Fig. 3:**
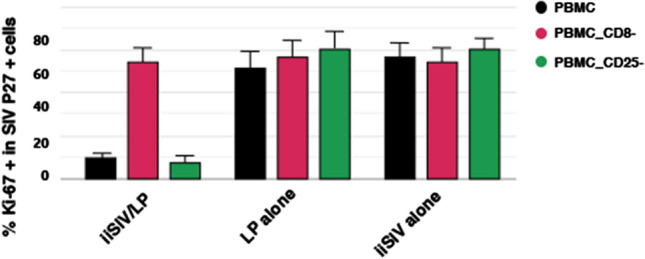
Percentage of activated cells (Ki-67^+^) among SIVp27^+^CD4^+^ T cells without or with depletion of CD8^+^ cells or CD25^+^ cells. In vaccinated macaques, SIV^+^CD4^+^ T cell activation was suppressed. Activation of SIVp27^+^CD4^+^ T cells was re-established when CD8^+^ T cells were withdrawn.

A central question was to understand how a mucosal vaccine administered to ChRMs, made of iSIV particles adjuvanted with BCG, LP or LR induced both the suppression of SIV-specific humoral and cellular responses and post-challenge sterile protection while the same vaccine administered without adjuvant induced a classical immune response and no post-challenge protection in ChRMs. We thus investigated whether vaccine-associated nonclassical CD8^+^ T cells could be involved in the suppression of SIV-specific CD4^+^ T cell activation (and thereby in the prevention of SIV infection). In a first set of experiments (Fig. 3), SIVp27^+^-prepared CD4^+^ T cells were stimulated overnight with SEB and anti-CD3/anti-CD28 antibodies and cultivated for five days in the presence or absence of autologous or allogeneic CD8^+^ T cells from vaccinated or control RMs. Because of the strong *in vitro* activation induced by SEB and anti-CD3/anti-CD28 antibodies, the percentage of activated (Ki-67^+^) SIVp27^+^CD4^+^ T cells was high (≥ 50%) in the absence of vaccine-induced CD8^+^ T cells or in the presence of CD8^+^ T cells from unvaccinated RMs; in contrast, in the presence of autologous CD8^+^ T cells from vaccinated RMs, the activation of SIV-CD4^+^ T cells was strongly suppressed, as shown by the percentage of Ki-67^+^SIVp27^+^CD4^+^ T cells, which dropped down to ≤10%. We also showed that vaccine-induced CD8^+^ T cells operated through a nonclassical MHC-restricted mechanism, since they suppressed the activation of SIVp27^+^CD4^+^ T cells from other vaccinated RMs as well as from control animals. Moreover, in the same set of experiments, we showed that the *ex vivo* removal of classical T-Regs (CD25^+^FoxP3^+^CD4^+^ T cells) by an anti-CD25 antibody did not modify the suppression of CD4^+^ T cell activation. Finally, using a highly sensitive cytotoxicity assay, we controlled the absence of CD4^+^ T cell lysis. This favors the conclusion that the suppression of SIV-specific CD4^+^ T cell activation is a non-cytotoxic process mediated by non-classical MHC-restricted CD8^+^ T-regulatory cells**.** We refer to these previously unrecognized cells as "tolerogenic CD8^+^ T cells".

### *Ex vivo* functional characteristics of vaccine-induced tolerogenic CD8^+^T-Regs

**Fig. 4:**
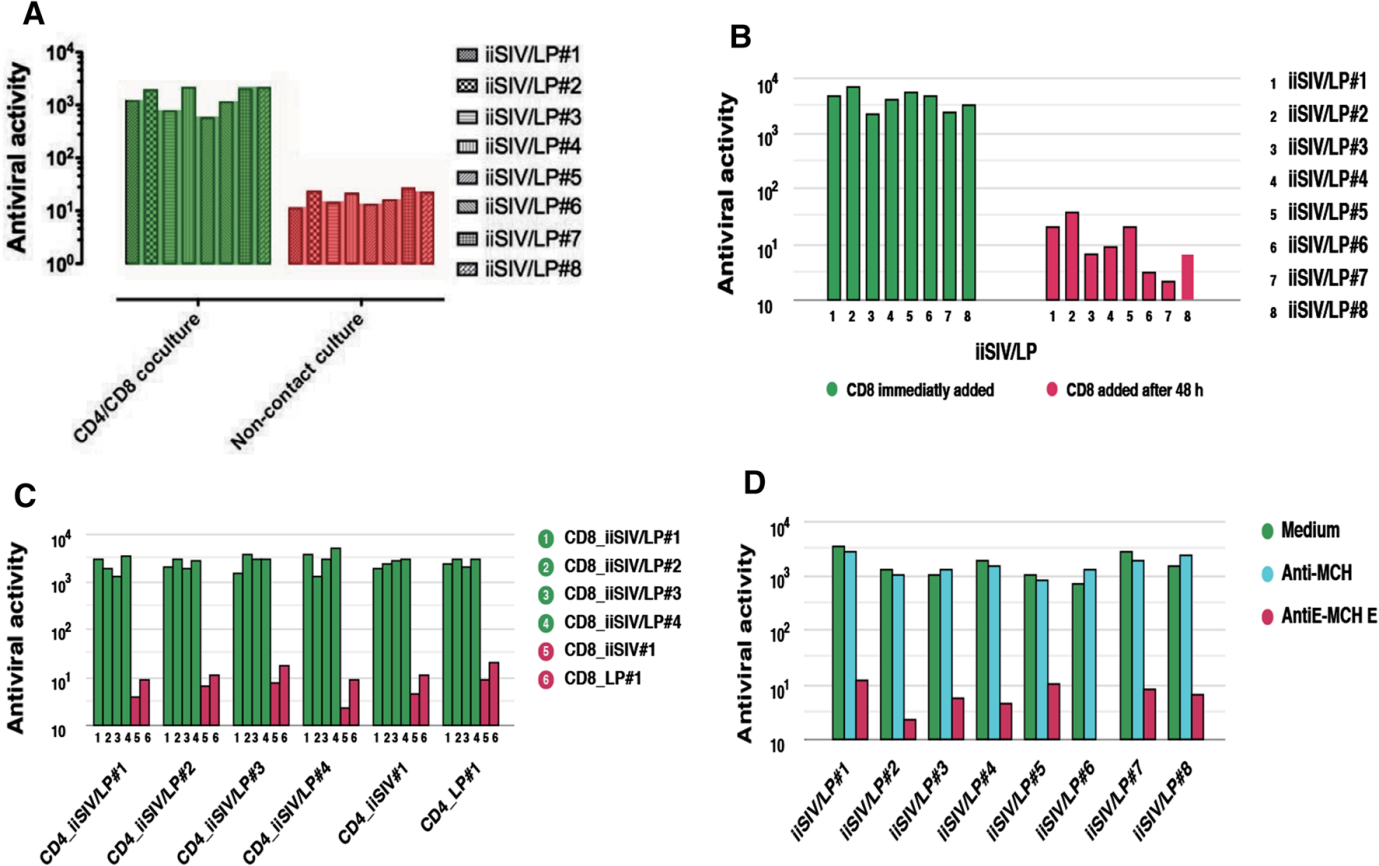
*Ex vivo* antiviral activity (SIV RNA suppression) of CD8^+^ T cells taken from vaccinated ChRMs. The data show the anti-SIV activity of CD8^+^ T cells 60 days after immunization in a delayed (A), insert (B), or allogenic (C) culture system in the presence of MHC-la/ABC or MCH-Ib/E antibodies (D).

Having demonstrated that adding LP (but also BCG or LR) to iSIV turned a classical SIV- specific immune response into an SIV-specific CD8^+^-T cell-dependent response characterized by the suppression of SIV-positive CD4^+^ T cell activation, we further showed that SIV-specific CD8^+^ T cells also prevented SIV replication in freshly infected CD4^+^ T cells from vaccinated ChRMs. When CD8^+^ T cells were added to the tube more than 48 hours after stimulation of SIV-infected CD4^+^ T cells (at a time when SIV-specific CD4^+^ T cell activation was already well established), CD8^+^ T cells could no longer inhibit viral replication, which clearly indicates that the action of vaccine-induced CD8^+^ T cells is a preventative one (and not a "therapeutic" one) (Fig. 4A). In the same set of experiments, we also demonstrated that the CD8^+^-T-cell-mediated antiviral activity required cell-to-cell contact (Fig. 4B) and was also MHC-unrestricted (Fig. 4C). Moreover, we showed that CD8^+^-T-cell-mediated antiviral activity was blocked by an anti-MHC-Ib/E antibody and not by an anti-Ia/ABC antibody, indicating a non-classical MHC-Ib/E-restricted CD8^+^ T cell activity (Fig. 4D). Overall, vaccine-induced CD8^+^ T cells prevented (most probably by contact) activation of SIV-RNA-infected CD4^+^ T cells, which in turn prevented the reverse transcription of SIV RNA into SIV DNA (which is activation dependent) and the subsequent cascade of events ending in virus replication and release. These findings are in keeping with the potential protective role of MHC-E observed in HIV-infected individuals [49].

### Predictive value of CD8^+^-T-cell-mediated antiviral activity in vaccinated macaques

**Fig. 5:**
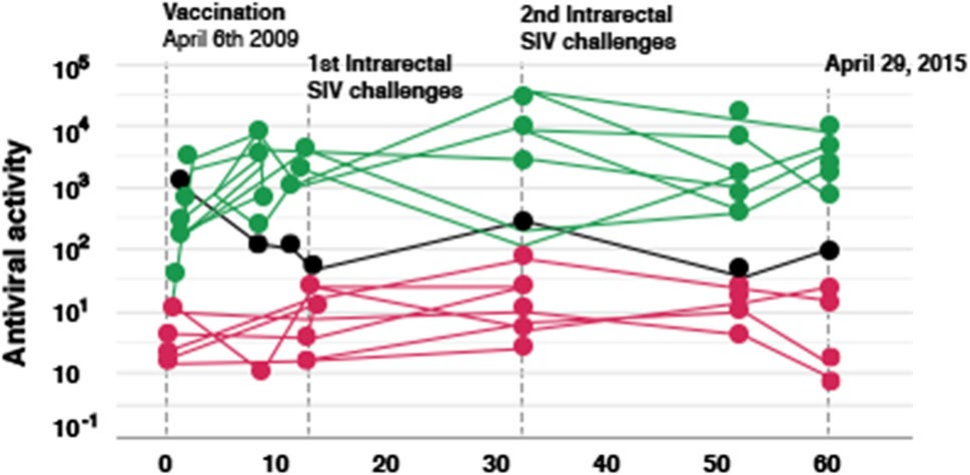
Antiviral activity (fold SIV suppression) of CD8^+^ T cells taken from vaccinated ChRMs

The antiviral activity generated by fresh tolerogenic CD8^+^ T cells was measured in iSIV+LP-vaccinated and control RMs. Out of the eight vaccinated RMs, seven were protected (see above); all seven showed strong pre-challenge antiviral activity, which remained stable until 5 years post-vaccination (Fig. 5). In contrast, the antiviral activity of CD8^+^ T cells from the only animal that was later shown to be not protected, increased until month 3 but then decreased until reaching a baseline level similar to those of unprotected control RMs. Altogether, the correlation between pre-challenge *ex vivo* antiviral activity of tolerogenic CD8^+^ T cells and post-challenge SIV protection suggested that the pre-challenge measurement of tolerogenic CD8^+^-T-cell-mediated antiviral activity could be a predictive marker of vaccine efficacy.

## Role of CD8^+^ T cells in the protection of macaques against intrarectal SIV challenge ***in vivo***

To confirm *in vivo* that CD8^+^ T cells were still capable of protecting ChRMs from an SIV challenge late (15 months) after vaccination, four RMs that were sterilely protected after three intrarectal challenges were given a fourth intrarectal infectious challenge. At the same time, the CD8^+^ T cells of these animals were depleted using an anti-CD8 antibody. No longer protected by their tolerogenic CD8^+^ T cells, all of the animals were soon fully infected; however, some weeks later, as soon as their tolerogenic CD8^+^ T cells recovered, the ChRMs again strongly controlled viral replication. However, recovered animals now contained SIV DNA in target cells, but without obvious viral replication.

## Similar tolerogenic CD8^+^ T cells in human elite controllers and vaccinated Chinese macaques

Having discovered that the suppression of viral replication in vaccinated ChRMs was resulting from vaccine-induced tolerogenic CD8^+^ T cells, the question we asked then was whether such tolerogenic CD8^+^ T cells, which were capable of protecting vaccinated ChRMs, might exist in human "elite controllers", the small percentage (<1%) of HIV-infected patients whose viral replication is naturally inhibited for long periods of time. Ten elite controllers were studied with the help of Chinese clinicians [6]. In nine of them (but zero of 10 HIV-1-infected patients with a high viral load), we identified a population of tolerogenic CD8^+^ T cells that had the same characteristics and displayed the same *in vitro* suppression mechanisms as those discovered in vaccinated ChRMs. By inhibiting the activation of HIV-infected CD4^+^ T cells, these human tolerogenic CD8^+^ T cells maintained the suppression of HIV replication over several years. The discovery of this tolerogenic CD8^+^ T cell population has clear implications for the design of an effective tolerogenic vaccine against HIV in humans.

## Conclusions

It is by serendipity that we discovered in 2006 a mucosal vaccine consisting of iSIV adjuvanted with BCG, and later on with LP or LR. This vaccine induced the suppression of SIV-specific immune responses and at the same time caused the activation of tolerogenic CD8^+^ T cells, a subset of CD8^+^ T cells that are SIV-specific, non-cytolytic and MHC-Ib/E-restricted. These tolerogenic CD8^+^ T cells have the ability to prevent the activation of SIV-RNA-infected CD4^+^ T cells and to maintain these cells in a quiescent state, thereby preventing the retro-transcription of SIV RNA into cellular SIV DNA, which arrests the infectious process and prevents the establishment of productive SIV infection both *in vivo* and *in vitro* (Table 1).

**Table 1:** Summary of immune-virological results in Chinese macaques immunized with inactivated SIV alone or with inactivated SIV adjuvanted with BCG, *Lactobacillus plantarum* or *L. rhamnosus*

	iSIV alone	iSIV + adjuvant
Activation of SIV-infected CD4^+^ T cells	Yes	Suppressed
Production of anti-SIV IgM and IgG	Yes	Suppressed
*Ex vivo* antiviral activity of CD8^+^ T cells	No	Yes
Sterile production after intrarectal homologous SIV challenge	No	Yes
Sterile production after intrarectal heterologous SIV challenge	No	Yes

Vaccine-induced tolerogenic CD8^+^ T cells have not been described previously in humans or RMs. However, the tolerogenic CD8^+^ T cells we discovered resemble those that targeted and eliminated abnormally activated antigen-specific CD4^+^ T cells in the mouse model [50, 51], where the inhibitory interaction depends on the recognition of surface Qa-1 (corresponding to MHC-IB/E in RMs and to HLA-E in humans) expressed by aberrantly activated target cells [52, 53]. The protective effect of this innovative vaccine, together with the identification of a correlate of protection *in vitro,* is quite striking. Given that SIV and HIV require activated immune cells in which to replicate, the specific prevention of activation of SIV-RNA-containing CD4^+^ T cells by a tolerogenic vaccine approach offers an exciting new avenue in HIV vaccine research.

Although the present formulation of the vaccine, which contains heat-inactivated virus, currently precludes a preventive vaccine trial in humans, we believe that the data are sufficiently impressive to justify a therapeutic trial in HIV-infected patients. Accordingly, we are currently seeking financial support to conduct a phase 1 trial on recently infected HIV carriers. The heat-inactivated HIV to be combined with *Lactobacillus plantarum* will be prepared separately under GMP conditions. The initial readout of this trial will be the induction of a population of tolerogenic (non-cytolytic) CD8^+^ T cells that have the ability to suppress CD4^+^ T cell activation by HIV *in vitro* (“surrogate” marker) in the way that has been described in monkeys [3, 6].

## Declarations

Some paragraphs in this review appeared in a previously published research article [54].
